# Regulation of autophagy by Ca^2+^

**DOI:** 10.1007/s13277-016-5353-y

**Published:** 2016-11-18

**Authors:** Fang Sun, Xia Xu, Xiaohong Wang, Bei Zhang

**Affiliations:** 1 Department of Clinical Medicine, Xuzhou Medical University, Xuzhou, Jiangsu 221000 China; 2 Department of Obstetrics and Gynecology, Xuzhou Hospital of Traditional Chinese Medicine, Xuzhou, Jiangsu 221002 China; 3 Department of Obstetrics and Gynecology, Xuzhou Central Hospital, Xuzhou, Jiangsu 221009 China; 4 Department of Obstetrics and Gynecology, Xuzhou Central Hospital, Xuzhou, Jiangsu 221002 China

**Keywords:** Ca^2+^, IP_3_Rs, Autophagic regulation, Apoptosis, Mitochondria

## Abstract

Autophagy is an evolutionarily conserved lysosomal catabolic process used as an internal engine in response to nutrient starvation or metabolic stress. A number of protein complexes and an intricate network of stress signaling cascades impinge on the regulation of autophagy; the mammalian target of rapamycin serves as a canonical player. Ca^2+^, as a major intracellular second messenger, regulates multiple physiological and pathological functions. Although significant information is already well-established about the role of Ca^2+^ in apoptosis, its role in autophagy has been recently determined and is poorly understood. Intracellular Ca^2+^ positively and negatively affects autophagy. In this review, evidence for both views and the interplay of Ca^2+^ between autophagy and apoptosis induction are discussed. The available data revealed the bidirectional role of Ca^2+^ in the regulation of autophagy. Moreover, the data also indicated that this role probably depends on the context of time, space, Ca^2+^ source, and cell state, thus either preventing or enhancing autophagy.

## Introduction

Autophagy is a main catabolic process of degrading and recycling intracellular components in lysosomes that cannot be executed by the ubiquitin–proteasome system. These components include long-lived proteins, damaged organelles, and some other macromolecules [1, 2]. Under well-fed conditions, autophagy in most cells is maintained at a low basal level, whereas under stressful conditions such as nutrition deprivation, high temperatures, oxidative stress, accumulation of damaged organelles, and cytosolic Ca^2+^ upload, autophagy is activated to play an essential role in sustaining cellular homeostasis and energy requirements, thus facilitating the survival of cells [3, 4]. Insufficient or altered autophagic flux is responsible for various diseases, such as cancer, neurodegenerative disorders, and cardiovascular disease, because of the significance of autophagy in cellular life and death processes [5, 6]. According to the manner of material delivery to the lysosomes, autophagy is divided into three main types, namely macroautophagy, chaperone-mediated autophagy, and microautophagy [7]. The former type is the most common form of autophagy (hereafter referred to as autophagy) and contains the formation and elongation of a typical double-membranous cistern (phagophores) into a whole vesicle (autophagosomes), which ultimately fuses with lysosomes to form autolysosomes, leading to the breakdown and recycling of the enclosed content [8].

The molecular basis of autophagy is complex. So far, more than 30 autophagy-related genes (ATGs) have been identified as crucial regulators of this complex process, from the initial signal to the final fusion [9–11]. In this family, one quite important member is Beclin 1, the mammalian ortholog of Atg6 in yeast; Beclin 1 plays a central role in the initial formation of autophagosome [12, 13]. Beclin 1 can dimerize and interact with Vps34, Vps15, Bif1, UVRAG, Ambra1, and some other proteins to generate phosphatidylinositol-3-phosphate (PI3P), which catalyzes vesicle elongation and phagophore nucleation by promoting the recruitment of other Atg proteins [14, 15]. Therefore, Beclin 1 depletion results in autophagic deficiency [16]. Remarkably, although the role of Beclin 1 in apoptosis has not been clearly elucidated, this ortholog has been determined as a member of the pro-apoptotic BH3-only protein family. Its BH3 domain mediates an interaction among anti-apoptotic Bcl-2 protein family members Bcl-2, Bcl-Xl, Mcl-1, and Bcl-w and thereby blocks the induction of the autophagic machinery under normal conditions [17, 18]. However, during autophagy-inducing conditions, Beclin 1 is allowed to dissociate from the Bcl-2–Beclin 1 protein complex and subsequently activates the PtdIns3K complex III to induce autophagy [19]. The interaction between Beclin 1 and Bcl-2 proteins can be dynamically regulated by various mechanisms. For instance, the phosphorylation of either Bcl-2 by c-Jun NH2-terminal kinase-1 or Beclin 1 by death-associated protein kinase (DAPK) antagonizes the interaction between the two proteins, and then, released Beclin 1 promotes autophagy [20, 21]. In addition, other proteins including BNIP3, nutrient-deprivation autophagy factor 1 (NAF-1), and high motility group box 1 (HMGB1) competitively disrupt the binding of Beclin 1 to Bcl-2 and regulate the onset of autophagy [22–24]. Furthermore, recent studies have shown that the suppression of Beclin 1’s function in autophagy is mainly attributed to Bcl-2 located at the endoplasmic reticulum (ER), with inhibitory effects on Ca^2+^-induced autophagy [25–27].

The network of stress signaling cascades and a number of protein complexes highlight significant considerations for autophagy regulation [28]. To date, the canonical and central sensor for the availability of energy and nutrients is the mammalian target of rapamycin (mTOR), in particular complex1 (mTORC1); mTOR functions as a negative regulator of autophagy [29, 30]. Although multiple signal pathways converge to modulate mTOR activity, one of the most important pathways is the AMP-activated protein kinase (AMPK), a positive regulator of autophagy [31]. Activated AMPK promotes the phosphorylation of the tuberous sclerosis complexes (TSC) 1 and 2. Subsequently, activation of TSC1/TSC2 suppresses mTOR activity by deactivating the mTORC1-interacting protein, Rheb, inducing autophagy [32–34]. In addition, AMPK can be phosphorylated and activated by Ca^2+^-calmodulin-dependent protein kinase kinase-β (CaMKKβ), which provides a close association among Ca^2+^ signaling, mTOR, and autophagy [35]. Indeed, recent studies have implicated the contributions of intracellular Ca^2+^ signaling and inositol 1,4,5-trisphosphate receptors (IP_3_Rs) to autophagy regulation; IP_3_Rs are tetrameric Ca^2+^ channels located at ER, and these channels release Ca^2+^ from the ER to the cytosol [36]. IP_3_Rs are major regulators of autophagy and apoptosis, which are also modulated by the members of the Bcl-2 family of proteins. The IP_3_Rs have been identified recently as a Bcl-2-regulated repressor of autophagy [37].

Ca^2+^, as a major intracellular second messenger, regulates multiple physiological functions in cells, such as contraction, secretion, metabolism, survival or death, and gene transcription. It also participates in some pathological processes [38, 39]. Previous studies showed that interference with calcium homeostasis can provoke cell death in many types of tumor cells [40]. A variety of cellular Ca^2+^-transporting and Ca^2+^-binding proteins are located mainly at the plasma, cytosol membrane, ER, and mitochondria, namely Ca^2+^ toolkit [41]. These correct spatiotemporal distributions of Ca^2+^ determine several of the most commonly recognized and well-studied intracellular signals [42, 43]. Although significant information is already well-established about the role of Ca^2+^ in apoptosis, the role of Ca^2+^ in autophagy regulation remains poorly understood. The role of Ca^2+^ signals involved in autophagy regulation was noted in the early 1990s, and the pioneering study already suggested a complicated role of Ca^2+^ as inhibitor and promoter of Ca^2+^ release from internal stores suppressed by autophagy [44]. In recent decades, a series of studies on the dual role of Ca^2+^ in autophagy regulation began to emerge, but their results still differ with respect to the precise mechanisms and pathways involved. On the one hand, numerous reports have revealed that Ca^2+^ functions as a negative regulator of autophagy [37, 45–50]. On the other hand, other studies indicated a stimulatory role of Ca^2+^ toward autophagy [25, 51–56]. The role of the ER as a physiologically important Ca^2+^ store is universally recognized, and IP_3_Rs may also have an important role in the control of autophagy, although the current data are at least partially contradictory [57, 58]. By releasing Ca^2+^ from the ER, these ubiquitously expressed channels control diverse cellular processes including cell proliferation and death. In addition, they play an essential role for a constitutive IP_3_R-mediated Ca^2+^ release to mitochondria in maintaining mitochondrial bioenergetics [48, 59–61].

In this study, the available evidence on the role of Ca^2+^ signal is summarized, in particular the IP_3_R-mediated Ca^2+^ release in the regulation of autophagy. Furthermore, the interplay of Ca^2+^ between autophagy and apoptosis induction is discussed. The available data not only showed a bidirectional role for Ca^2+^ in the control of autophagy but also suggested a model showing that this role may rely on the specific context, thus either preventing or enhancing autophagy.

## Inhibition of autophagy by IP_3_R/Ca^2+^

An initial study indicating the inhibitory effect of IP_3_R on autophagy was based on the use of lithium (Li^+^). Li^+^-stimulated autophagic response, in turn, results in declined IP_3_ levels and diminishing Ca^2+^ release from the ER, which then triggers autophagy [45]. The study revealed a novel mTOR-independent manner that regulates autophagy. Criollo et al. subsequently confirmed IP_3_R in the control of autophagy [37]. They showed that chemical blockade with xestospongin B (XeB) or the silence of the IP_3_R with small interfering RNAs is a strong stimulus for the induction of autophagy in HeLa cells. They also reported an organelle-specific interaction between IP_3_R and Bcl-2 and proposed that IP_3_R may function as a Bcl-2-regulated inhibitor of autophagy, which specifically targeted the ER but not the mitochondria. However, ER stress-activated autophagy is not suppressed by Bcl-2, which implies a variety of signaling pathways involved in the regulation of autophagy. In view of the uncertain link between Ca^2+^ signaling and IP_3_R-modulated autophagy, the same laboratory proceeded to investigate and propose that IP_3_R-mediated Ca^2+^ release may not be an entitative part of the mechanism, whereas protein interactions with the channel may play a dominant role [46]. Thus, they presented evidence showing that IP_3_R may shut down autophagy by decreasing the release of Beclin 1 from Bcl-2-mediated sequestration, therefore facilitating the formation of anti-autophagic Bcl-2–Beclin 1 complexes. Moreover, xestospongin B or nutrient starvation would disrupt the interaction between the IP_3_R and Beclin 1 and thus release Beclin 1 available for the induction of autophagy. In this model, although autophagy negatively modulated by IP_3_R may be attributed to the obligate contribution of Beclin 1 instead of the involvement of the IP_3_R Ca^2+^ channel function, the IP_3_R was identified as an unsuspected regulator of the Beclin 1 complex and bridged an intriguing signal network that converges on the ER and initial phagophore formation.

The explicit mechanism of IP_3_R in autophagy regulation remains to be explored. Other studies did not confirm that the modulation of autophagy by the IP_3_R was independent of IP_3_-induced Ca^2+^ release. Glucocorticoids were shown to attenuate IP_3_-dependent calcium signaling and then induce autophagy by downregulating the src kinase Fyn, which is identified as a positive regulator of IP_3_-mediated calcium release by phosphorylating type I IP_3_ receptors (IP_3_R1) at Tyr353. Here, the induction of autophagy appeared to involve the canonical mTOR pathway [49]. Several groups investigated the role of IP_3_R using a unique IP_3_R-null cell line, DT40 chicken B cell lines, in which all three IP_3_R isoforms were genetically deleted (triple knockout (TKO) cells). Using this experimental model, Khan et al. observed a markedly elevated basal autophagic flux in TKO cells compared with wild-type cells even under nutrient-fertile conditions. They demonstrated that the Ca^2+^ channel function of the IP_3_R was essential for autophagy inhibition by IP_3_R. Furthermore, they documented that IP_3_R-mediated Ca^2+^ signals could regulate the autophagic pathway attributable to the inhibition of mTORC1 activity rather than correlated with AMPK, Akt, or Bcl-2–Beclin 1 complexes [47]. Similarly, Cardenas et al. reported higher levels of autophagic markers in TKO DT40 than their wild-type counterparts because of the absence of IP_3_R Ca^2+^ release activity; although in their study, they attributed this effect to reduced ATP production and the activation of AMPK but not mTOR [48]. By contrast, another study reported no evident difference between autophagy in wild-type and TKO DT40 cells presumably because of an adaptive alteration of the TKO DT40 cells and the expression of a truncated version of IP_3_R [46]. Although the basis for these discrepant results remains unknown, the existence of multiple regulatory mechanisms is not remarkable in the view of the complexity of the autophagic pathway and the differences in cell growth stage and stimuli.

A detailed explanation of the inhibitory effect of IP_3_R on autophagy induction was clarified by Foskett et al. [48], who reported the involvement of mitochondrial performance. This phenomenon is based on the presence of IP_3_Rs in ER domains, which are sensed by nearby mitochondria and permit the efficient transfer of Ca^2+^ from the ER to the mitochondria. This study showed that the impairment of IP_3_R-mediated Ca^2+^ release activity in TKO cells results in diminished Ca^2+^ uptake by the mitochondria. Subsequently, the diminished Ca^2+^ uptake results in decreased O_2_ consumption, reduced ATP production, and activation of AMPK, which indicate that mitochondrial oxidative phosphorylation was constitutively compromised. This compromise activates pro-survival autophagy even in nutrient-replete media. In addition, the researchers identified a molecular mechanism for autophagy regulation that involved non-canonical AMPK-dependent pathway because mTOR activation seemed to be unaltered. These observations suggest that constitutive IP_3_R-mediated Ca^2+^ release to the mitochondria is fundamentally essential for efficient mitochondrial respiration, maintenance of optimal cellular bioenergetics, and suppression of autophagy. Subsequent studies showed that the ER membrane protein Bax inhibitor-1 (BI-1) overexpression suppresses IP_3_R-dependent Ca^2+^ delivery from the ER to the mitochondria and thereby affects mitochondrial bioenergetics and facilitates autophagy [62]. The results identified BI-1 as a novel autophagy regulator that bridges Ca^2+^ signaling between the ER and the mitochondria through a mechanism that decreases cellular oxygen consumption and promotes AMPK activation. Consequently, this regulator contributes to cellular resilience in response to metabolic stress.

**Fig. 1:**
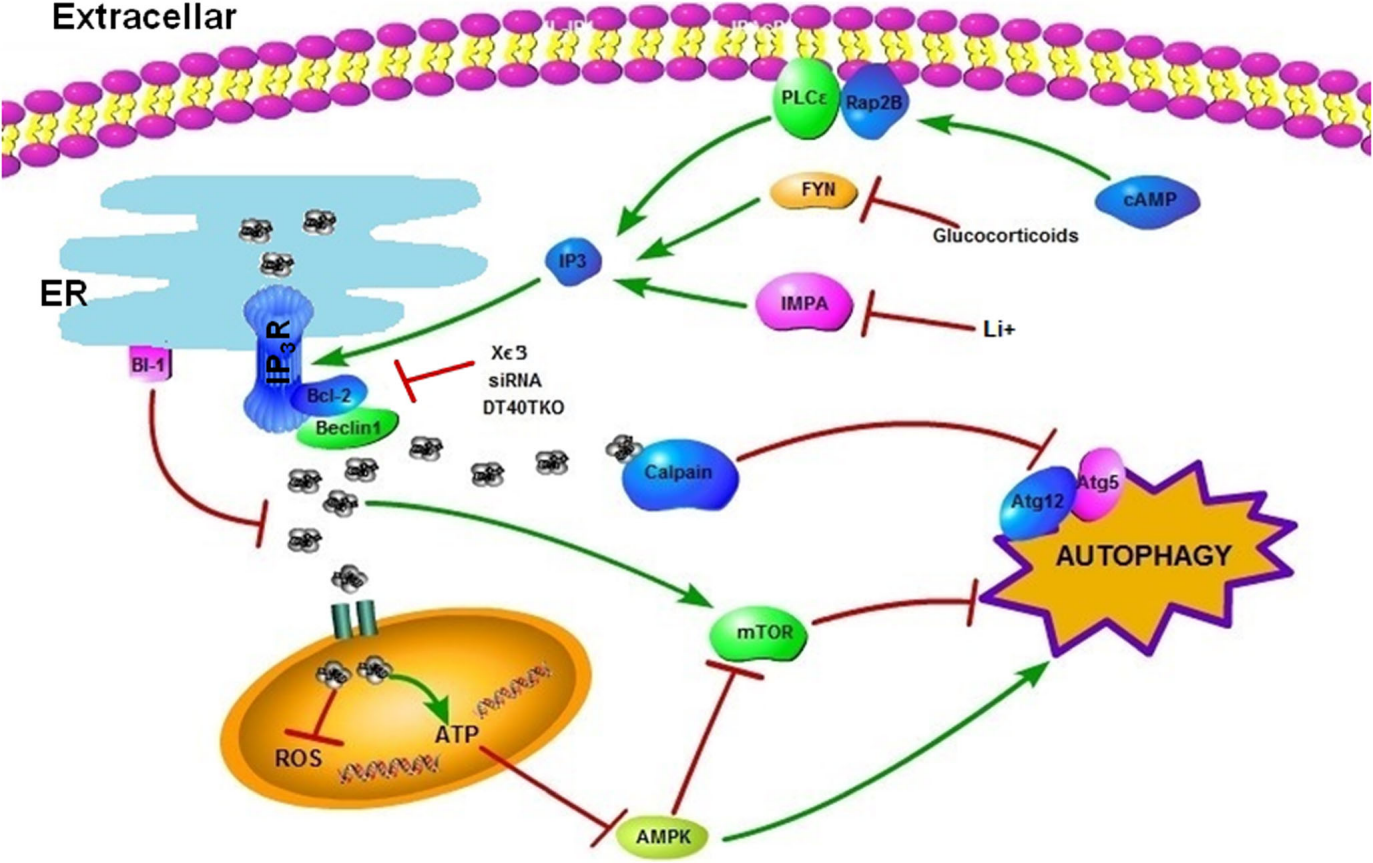
Ca^2+^ inhibits autophagy. On one hand, IP_3_R-mediated Ca^2+^ release toward the mitochondria results in increased ATP production and the suppression of AMPK and then inhibits autophagy. However, on the other hand, someone has attributed this phenomenon to the regulation of mTORC activity by Ca^2+^ instead of correlating with AMPK. Therefore, the inhibition or downregulation of IP_3_R with xestospongin B, siRNA, or in DT40 triple knockout (TKO) cells will promote autophagy. ER membrane protein Bax inhibitor-1 (BI-1) could suppress IP_3_R-dependent Ca^2+^ delivery from ER to mitochondria, thereby affecting mitochondrial bioenergetics and facilitating autophagy. IP_3_R might shut down autophagy by decreasing the release of Beclin 1 from Bcl-2-mediated sequestration, therefore facilitating the formation of anti-autophagic Bcl-2–Beclin 1 complexes. In addition, the IP_3_R-induced exit of Ca^2+^ from the ER is sufficient in activating calpains, which then cleave Atg5, leading to the inhibition of autophagy by decreasing the levels of the Atg5–Atg12 complex. This effect was shown to be well-regulated by the cAMP–Rap2B–PLCε pathway. Glucocorticoids induce autophagy by downregulating Fyn, which can promote IP_3_-mediated calcium release. Reducing IP_3_ levels through the addition of Li^+^, which inhibits IMPase, therefore induces autophagy

Autophagy induction can be mediated by L-type Ca^2+^ channel blockers, a K^+^_ATP_ channel opener, and Gi signaling activators, although they do not act directly on IP_3_R [50]. The latter data revealed an mTOR-independent pathway regulating autophagy, in which the established pathway from cyclic adenosine monophosphate (cAMP) to IP_3_ was identified as a strong negative regulator of autophagy by increasing intracytosolic Ca^2+^ levels and influencing calpain activity, indicative of a positive feedback loop of intracellular Ca^2+^ on autophagy inhibition. The IP_3_R-induced exit of Ca^2+^ from the ER leads to an increase in intracytosolic Ca^2+^, which is sufficient to activate the calcium-dependent cysteine protease calpains. Elevated LC3-labeled autophagosomes were observed after treatment with calpain inhibitors or transfection with siRNA of either calpain 1 or calpain 2. Similarly, Yuan et al. conducted a study using flurispirene, which is a compound that can block IP_3_-mediated Ca^2+^ release and activates autophagy [63]. Furthermore, these authors proposed a mechanism by which decreased intracellular Ca^2+^ prevents the calpain 1-mediated cleavage of Atg5, which, in turn, elevates the levels of the Atg5–Atg12 complex, necessary for the induction of autophagy. In agreement with the abovementioned studies, Mestre et al. showed that the deactivation of calpains by their inhibitor calpeptin could allow autophagy activation and revert cAMP inhibition of the autophagy induced by the toxin, thus confirming the negative effects on the autophagy regulation of calpains [64]. An overview of the inhibitory pathways described in this section is presented in Fig. 1.

## Induction of autophagy by Ca^2+^

In contrast to the inhibitory roles of IP_3_R/Ca^2+^ in autophagy regulation, numerous experimental conditions that consider Ca^2+^ as an activator of autophagy have also emerged in recent decades. However, the physiological relevance of many of these observations is ambiguous, and the precise mechanisms involved are always debatable. Hoyer-Hansen et al. provided a link among Ca^2+^ signaling, mTOR, and autophagy in detail [25]. They eventually demonstrated that elevated cytosolic calcium concentration ([Ca^2+^]_c_) by Ca^2+^-mobilizing agents functions as a potent trigger of autophagy through a mechanism that involves CaMKKβ, AMPK, and the subsequent suppression of mTOR signaling. This process could be inhibited by ER-located Bcl-2, which effectively lowers [Ca^2+^]_ER_ and attenuates agonist-induced Ca^2+^ fluxes. Similarly, the latter data showed that the increase in [Ca^2+^]_c_ by other pharmacological agents can also activate autophagy through the CaMKKβ–AMPK–mTOR pathway [56, 65], which has vital roles in diverse cellular processes including ribosome biogenesis and transcription in addition to metabolism and cell motility [66]. Furthermore, amyloid-β protein promotes the formation of autophagic vesicles also through the same CaMKKβ and AMPK signaling [67].

In addition to the “canonical” CaMKKβ–AMPK–mTOR pathway, Grotemeier et al. used an AMPK-independent pathway to account for Ca^2+^-activated autophagy. In their study, thapsigargin treatment, as a result of the elevation of cytosolic Ca^2+^, triggers autophagy in the absence of AMPK, with only minimal changes noted in mTOR activity. Thapsigargin, a non-competitive Ca^2+^ ATPase, can increase intracellular calcium by blocking the cell’s ability to pump calcium into the sarcoplasmic and endoplasmic reticula. The researchers further concluded that Ca^2+^ signaling triggering autophagy could be AMPK-dependent or AMPK-independent, and the inhibition of CaMKKβ reduces the level of thapsigargin-induced autophagy only in AMPK-positive cells [68]. Sakaki et al. showed another mechanism without inhibiting mTOR activity in thapsigargin-induced autophagy, but protein kinase C θ (PKCθ) activation followed by elevation in [Ca^2+^]_c_ is specifically required for ER stress-induced autophagy, which could be blocked by the chelation of intracellular Ca^2+^ with BAPTA-AM [52]. In addition, another group demonstrated that Ca^2+^ release from the ER after cadmium treatment induces autophagy through the calcium–ERK pathway [53], although the targets of ERK were not clearly elucidated. Therefore, ERK may be associated with the phosphorylation of Bcl-2, thereby resulting in Beclin 1 release from Bcl-2 [69]. Following up on these studies, a recent investigation documented that CaMKI promotes the formation of autophagosomes through a pathway that involves PtdIns3K complex III instead of depending on AMPK (32). In this regard, Vps34, a component of the PtdIns3K complex III, is activated by calmodulin and Ca^2+^ [70].

Moreover, the overexpression of leucine-rich repeat kinase-2 (LRRK2) promotes the release of Ca^2+^ from lysosomal stores in human embryonic kidney cells, which, in turn, causes a persistent increase in autophagic vesicle formation through the Ca^2+^/CaMKKβ/AMPK pathway, but this effect of LRRK2 on autophagy induction is found to be mTORC1-independent [71]. In addition, LRRK2 overexpression upregulates the levels of SQSTM1/p62 in a Ca^2+^-dependent manner, which links ubiquitinated targets to the autophagic machinery [72]. Finally, a recent study inferred that quercetin could induce autophagy and is attributed to the elevated Ca^2+^, although the specific mechanisms of the calcium-signaling pathway in quercetin-induced autophagy remain to be revealed [73].

Recent studies exposed the presence of calcium microdomains mediated by different calcium channels situated at different cellular compartments, namely sarcolemma, mitochondria, sarcoplasmatic reticulum, and lysosome [74]. TRPML3, a Ca^2+^-permeable channel expressed in many intracellular compartments, including endosomes, lysosomes, and autophagosomes, is implicated in autophagy regulation. By specifically interacting with the mammalian Atg8 homolog GATE16, TRPML3 facilitates autophagosome maturation by providing Ca^2+^ in the fusion process [75]. Medina et al. showed that local calcineurin activation can occur in the vicinity of the lysosome through the lysosomal Ca^2+^ channel MCOLN1 and demonstrated that lysosomal Ca^2+^ signaling could induce autophagy by activating the phosphatase calcineurin and its substrate TFEB, which is a master transcription factor of lysosomal biogenesis and autophagy. They analyzed a Ca^2+^-dependent pathway that originates from the lysosome and modulates autophagy at the transcriptional level [76]. Furthermore, the knockdown or overexpression of the plasma membrane Ca^2+^ channel MCOLN3 can inhibit or activate autophagy, respectively. Remarkably, MCOLN3 is also recruited for autophagic vesicles upon the activation of autophagy, implying the important role of Ca^2+^ in autophagosome–lysosome fusion [77].

In addition to the treatment of Ca^2+^-mobilizing agents or the sarcoplasmic/endoplasmic reticulum Ca^2+^ ATPase (SERCA) inhibitors directly or indirectly leading to an elevated [Ca^2+^]c and subsequently stimulating autophagy, other autophagy inducers (such as starvation and rapamycin) may also result in autophagy stimulation through enhanced Ca^2+^ signaling, which could be blunted by the Ca^2+^ chelator BAPTA, as well as by the anti-apoptotic protein Bcl-2 [26]. Moreover, PK11195, an established chemosensitizer of tumor cells, functions as a facilitator of Ca^2+^-mediated autophagy by targeting the Bcl-2, which in its active form can decline [Ca^2+^]_ER_. Thus, PK11195 inhibits stimulus-induced Ca^2+^ release and Ca^2+^-mediated autophagy [78]. As Bcl-2 can inhibit IP_3_-mediated Ca^2+^ release, it may evoke dispute that IP_3_R sensitization by Beclin 1 is due to its effects on Bcl-2 by dissociating Bcl-2 from IP_3_Rs. However, a Beclin 1 mutant could sensitize IP_3_-induced Ca^2+^ release, even if it had failed to bind Bcl-2 [79], suggesting that these events were not only dependent on the suppression of the inhibitory effect of Bcl-2. Similarly, a recent study showed that an increased IP_3_R-mediated Ca^2+^ delivered from the ER and a sensitization of IP_3_R were observed in starved cells, which were required for starvation-induced autophagy. In this process, Beclin 1 was essential for IP_3_R sensitization, independent of its ability to bind Bcl-2 but rather due to its intrinsic property to bind IP_3_R [80]. A related study by Ghislat et al. demonstrated that starvation induces autophagy partly through elevated levels of cytosolic Ca^2+^ that activates the CaMKKβ–AMPK–mTOR pathway, subsequently resulting in ULK1 stimulation. ULK1 is a significant protein in autophagy and is a part of the ULK1 complex, which is necessary in the early stages of autophagosome biogenesis [81].

**Fig. 2:**
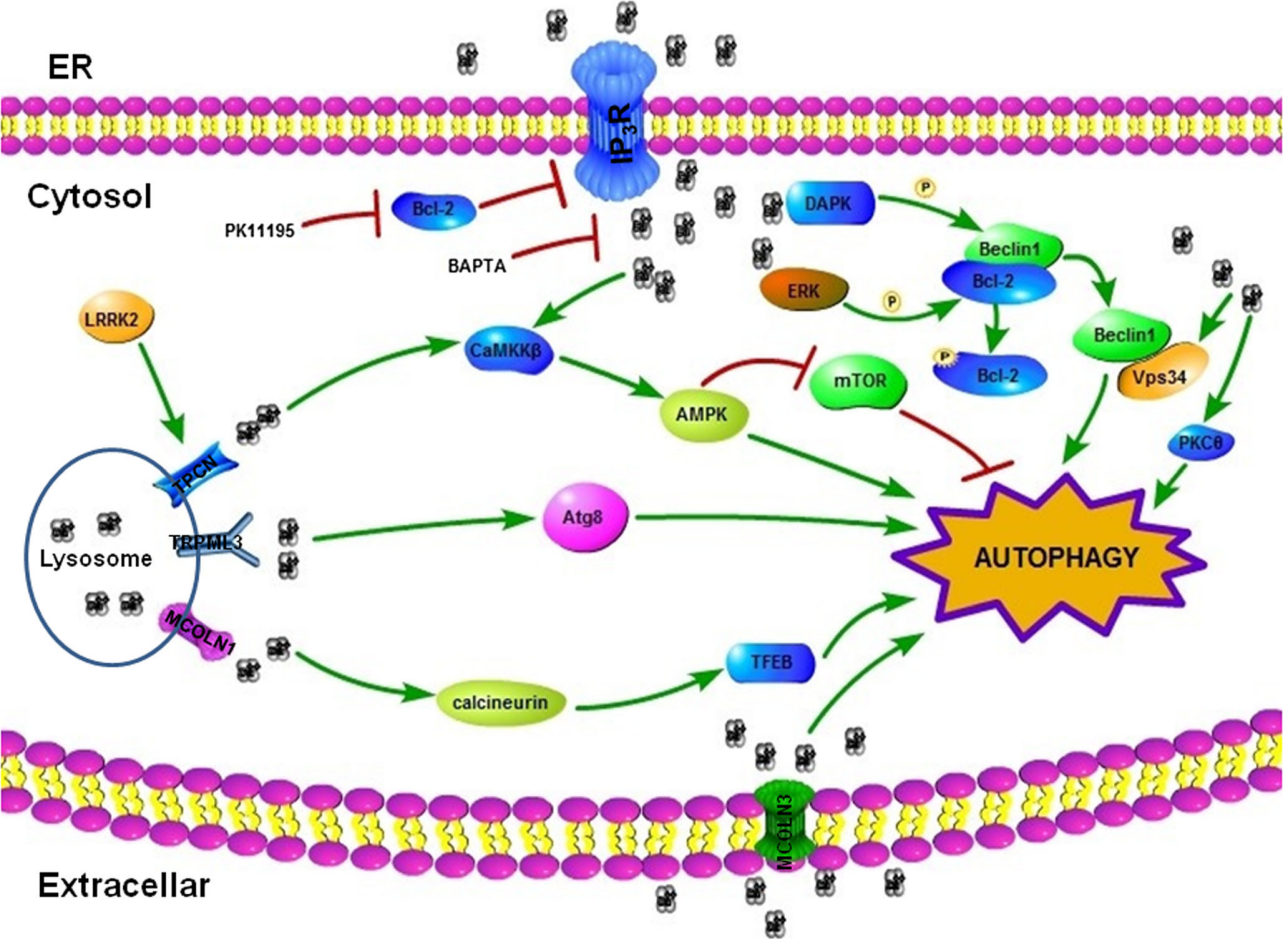
Ca^2+^ induces autophagy. The cytosolic calcium concentration can be elevated by multiple factors, including Ca^2+^-mobilizing agents, the sarcoplasmic/endoplasmic reticulum Ca^2+^ ATPase (SERCA) inhibitors, or other autophagy inducers (such as starvation and rapamycin). These factors stimulate IP_3_R-mediated Ca^2+^ release from the ER. Stimulus-induced Ca^2+^ release could be blunted by the Ca^2+^ chelator, BAPTA, or Bcl-2. Increased cytosolic Ca^2+^ can trigger autophagy through various mechanisms; the activation of CaMKKβ by Ca^2+^ stimulates the AMPK-mediated suppression of mTOR signaling. The overexpression of LRRK2 promotes Ca^2+^ release from lysosomal stores and then initiates autophagy through the Ca^2+^/CaMKKβ/AMPK pathway, but this effect is mTOR-independent. Ca^2+^-activated autophagy can also be AMPK-independent. PKCθ activation followed by elevated Ca^2+^ is required for ER stress-induced autophagy. Ca^2+^-ERK pathway is also involved in autophagy induction, which may be associated with the phosphorylation of Bcl-2, thereby resulting in Beclin 1 release from Bcl-2. In addition, Ca^2+^ can activate DAPK, which phosphorylates Beclin 1, thereby mediating its dissociation from Bcl-2. Vps34 may also be activated by Ca^2+^, although this activation is still debated. Lysosomal Ca^2+^ signaling could also induce autophagy. TRPML3, a Ca^2+^-permeable channel mainly expressed in lysosomes, facilitates autophagosome maturation by providing Ca^2+^ in the fusion process through a specific interaction with Atg8. MCOLN1 and lysosomal Ca^2+^ channel could induce autophagy by activating the calcineurin and its substrate TFEB. Furthermore, the plasma membrane Ca^2+^ channel MCOLN3 also activates autophagy

To date, although different mechanisms have already been proposed to account for Ca^2+^-mediated autophagy activation, this activation may be more complicated and depend on one or more of these mechanisms. Several other Ca^2+^-dependent targets are present in the regulation of autophagy, including the calmodulin-dependent DAPK, which positively regulates autophagy in several ways [82]. One way is the phosphorylation of Beclin 1 in its BH3 domain, which will finally promote the dissociation of Beclin 1 from Bcl-2/Bcl-xL-mediated sequestration [83]. Another approach is Ca^2+^ targets involved in autophagy activation that are the members of the S100 Ca^2+^-binding protein family, which include the S100B and S100A8/A9 complexes [84, 85]. S100B could interact with and then activate inositol monophosphatase (IMPase), which plays an important role in increasing IP_3_ production and subsequent Ca^2+^ release, constituting an amplification loop in the context of autophagy regulation. An overview of the stimulative pathways described in this section is presented in Fig. 2.

## Ca^2+^ connection in autophagy and apoptosis

Autophagy plays a protective role in maintaining cell survival during nutrient deprivation or other stressful situations, whereas cells undergo apoptosis when autophagy is suppressed [3, 86]. The inhibition of autophagy in HepG2 cells has pro-apoptotic effects, partly through enhanced ROS generation and activation of the mitochondrial apoptotic pathway, although the exact function of autophagy in apoptosis remains to be studied further [87]. Recent reports revealed that oxidative stress-triggered autophagic cell death is independent of apoptosis [87], suggesting that the processes of apoptosis and pro-survival autophagy are interrelated in a complicated manner or occur independently of each other. Therefore, the interplay between autophagy and apoptosis is controversial, and the scenario of autophagy in deciding cell destiny is elusive. At the molecular level, the cross talk between autophagy and apoptosis is beginning to emerge, and some autophagy-related and apoptosis-related proteins are identified as common regulators of both pathways, including the Beclin 1/Bcl-2 interaction. In addition, the tumor suppressor p53 is involved in autophagy regulation in addition to its pro-apoptotic effects [6]. Furthermore, many Atg proteins are caspase substrates, which gain pro-apoptotic functions through the cleaved C-terminal fragment of Beclin 1 [88, 89]. A similar switch from pro-autophagic to pro-apoptotic effects were represented on the calpain-mediated Atg5 cleavage [90], clearly indicating many potential links between these two pathways.

In particular, Ca^2+^ is a well-recognized regulator of multiple intracellular processes including apoptosis and autophagy. Therefore, the Ca^2+^ channel, IP_3_R, plays a crucial role [41]. Most of the compounds applied for Ca^2+^-mediated autophagy also promote apoptosis [25, 65, 78]. Thus, one might speculate that Ca^2+^ primarily activates apoptosis followed by the activation of autophagy [53]. However, this hypothesis is refuted by several data. The induction of autophagy occurs differently from apoptosis during cadmium treatment, and variations of Ca^2+^ concentration in cells seem to play a key role in regulating apoptosis and autophagy. This study demonstrated calcium-ERK-mediated autophagy and calcium-mitochondria-caspase-induced apoptosis [53], strongly suggesting that autophagy and apoptosis are Ca^2+^-dependent but go through different pathways. The differences between autophagy and apoptosis induction were also mentioned with regard to thapsigargin, ionomycin, and vitamin D3 [25]. However, different from cadmium-induced cytotoxicity, the induction of autophagy by quercetin manifests a protective effect by inhibiting apoptosis, attenuating lipid peroxidation, and recovering mitochondrial function [73]. Furthermore, recent evidence has shown that cells undergo autophagy instead of apoptosis if Bax/Bak is knocked out after hypericin-PDT treatment, which leads to ER Ca^2+^ depletion, although whether this phenomenon arises from the destruction of Ca^2+^ homeostasis or the subsequent ER stress was not investigated [51]. In addition, Lam et al. reported that Dictyostelium, an organism that cannot experience apoptosis, leads to autophagic cell death through IP_3_R, Ca^2+^ fluxes, and Ca^2+^-related protein [55].

Autophagy and apoptosis are related to the Ca^2+^ signal pathway. Therefore, confirming both outcomes during the particular treatment and reaching optimal conditions for the activation of autophagy but not for the induction of apoptosis are of great significance. The two outcomes are probably attributed to the different intracellular Ca^2+^ signals, which depend on the cellular location and the strength of the signal. Locally, cytosolic Ca^2+^ signals probably stimulate autophagy, whereas an elevated mitochondrial Ca^2+^ concentration can promote apoptosis [60]. In consideration of strength, Ca^2+^ oscillations may facilitate mitochondrial bioenergetics, but excessive Ca^2+^ transients in the mitochondria may result in permeabilization transition pore opening and subsequent mitochondrial outer membrane permeabilization.

## Conclusions

The findings presented in this review provide novel insights into IP_3_R and Ca^2+^ in autophagy regulation in addition to their explicit participation in apoptosis. Nevertheless, the regulation of autophagy by Ca^2+^ is very plausible, and the exact mechanisms are still under debate. Stimulatory as well as inhibitory roles for Ca^2+^ toward autophagy have been proposed, depending on the cell state and reflecting different spatiotemporal Ca^2+^ signals in unstressed versus stressful situations. Under normal conditions, constitutive IP_3_R-mediated Ca^2+^ signals arise from the ER into the mitochondria for a certain level of ATP production. A high ATP/AMP ratio is sufficient in deactivating AMPK and therefore shutting down the induction of autophagy. The suppression of this signal leads to the activation of autophagy because of the aberrantly insufficient energy production. As a consequence, unstressed cells exhibit an autophagy restraining Ca^2+^ signal, which specifically targets mitochondria bioenergetics and underpins a crucial role for IP_3_R-induced Ca^2+^ release in the typical microdomains. However, when cells encounter stressful conditions, Ca^2+^ signaling is intensified, and an elevated Ca^2+^ level is required for autophagy induction. In this way, the downstream targets of Ca^2+^ may be cytosolic and not confined to a specialized microdomain. Although the exact pathway has not been completely understood, we can speculate that either CaMKKβ–AMPK–mTOR pathway or AMPK-independent way is involved. Generally, cells can switch their Ca^2+^ signal from an “unstressed” autophagy-suppressive and mitochondrial signal to a “stressful” autophagy-stimulative and cytosolic signal. Irrespective of the underlying mechanism, this bidirectional regulation of autophagy by the IP_3_R and Ca^2+^ plays an important role in determining cell fate. As autophagy is closely involved in pathological situations including cancer and neurodegenerative diseases, the correct understanding of the relationship between autophagy and Ca^2+^ dynamics may shed light on important therapeutic strategies.
